# A multicenter cross‐sectional study of episiotomy practice in Romania

**DOI:** 10.1111/jep.13062

**Published:** 2018-11-13

**Authors:** Andrada Pasc, Dan Navolan, Lucian Pușcașiu, Cringu Antoniu Ionescu, Florin Adrian Szasz, Adrian Carabineanu, Mihai Dimitriu, Daniel Călin, Roxana Bohilțea, Liana Ples, Dragoș Nemescu

**Affiliations:** ^1^ Department of Obstetrics Gynecology University of Medicine and Pharmacy Targu Mureș Romania; ^2^ Department of Obstetrics Gynecology “Victor Babes” University of Medicine and Pharmacy Timisoara; ^3^ Help Prevent Foundation For Promotion Of Prevention and Health Resița Romania; ^4^ Department of Obstetrics Gynecology “Carol Davila” University of Medicine and Pharmacy Bucharest Romania; ^5^ Department of Obstetrics Gynecology University of Medicine and Pharmacy Oradea Romania; ^6^ Department of Surgery Victor Babeș University of Medicine and Pharmacy Timișoara Romania; ^7^ Department of Obstetrics Gynecology and Neonatology “Gr. T. Popa” University of Medicine and Pharmacy Iasi Romania

**Keywords:** episiotomy, episiotomy rate, Romania, vaginal birth

## Abstract

**Rationale, aims, and objectives:**

The aim of this study was to focus attention on episiotomy practice in Romanian maternity units in order to identify factors associated with the very high rate of the procedure in Romania and to consider strategies to reduce it.

**Methods:**

In this clustered cross‐sectional study, a total of 11 863 patients were recorded in eight Romanian maternity units to assess the prevalence of episiotomy. A random effects Poisson model was used to estimate the prevalence rate in univariate and multivariate models.

**Results:**

Among the 11 863 patients included for analysis, 8475 (71.4%) had an episiotomy. The prevalence of episiotomy was 92.7% for the first vaginal birth, 73.2% for the second vaginal birth, and 35% for the third vaginal birth. The overall rate of suturing was higher than the episiotomy rate for all patients (total rate 79.2%). The likelihood of exiting the maternity ward with an intact perineum after the first vaginal birth was less than 5% at the first vaginal birth.

**Conclusions:**

In conclusion, routine episiotomy is the norm in Romanian maternity units, with episiotomy rates among the highest in Europe. Episiotomy use is mainly driven by local professional norms, experiences, previous training, and practitioners' decisions rather than evidence, guidelines, or variations in patient needs at the time of vaginal birth.

**Abbreviations**
ICC: intra‐class correlation coefficient
PR: prevalence ratio

## 1 INTRODUCTION

Episiotomy is a surgical cut of the perineum performed in the second stage of labor in order to facilitate the birth of an infant by enlarging the vaginal opening.^1^ Episiotomy became a routine practice well before research results were available to support it. Two Cochrane Reviews in 2009 and 2017 pointed out that restrictive use of episiotomy was associated with a lower risk of clinically relevant morbidities including posterior perineal trauma, need for suturing perineal trauma, and healing complications.^2^, ^3^ Despite decades of research, which many interpret as having provided definitive evidence against the routine use of episiotomy, little professional consensus has been reached about the suitability of routine use.^4^

The rates of episiotomy in Europe are wide ranging, spanning 3.7% in Denmark to 75.0% in Cyprus.^5^ In a multicenter retrospective study conducted between 2003 to 2005 (Period 1) and 2012 to 2014 (Period 2), performed in Burgundy, France, the overall episiotomy rate reduced from 35.8% to 16.7%.^6^ In North America, a 17% decrease in episiotomy rate from 46.9% to 38.8% was achieved in the year 2006 after the introduction of a physician educational program,^7^ whereas another report found that the episiotomy rate ranged from 6.7% to 22.9% in operative vaginal deliveries in 2016.^8^

In many countries, including Romania, a number of obstetric health care practitioners consider that episiotomy should be used to prevent perineal trauma, pelvic floor relaxation, and its consequences, such as bladder prolapse and urinary incontinence. Furthermore, some practitioners prefer episiotomy because it is easier to repair than the laceration that results when episiotomy is not used.^9^ Simultaneous belief in the prevention of future sequelae and ease of repair creates the potential for misattributed motivations.^4^

The present study aimed to assess the prevalence of episiotomy in Romania, to identify factors associated with the practice of episiotomy, and to suggest strategies to reduce this practice in maternity units.

## 2 METHODS

### 2.1 Study design

Data were collected from the maternity wards of eight Romanian hospitals from September through December 2013. All singleton vaginal births (live births and stillbirths) that occurred during this period were included. Information was extracted from obstetric and neonatal records.

### 2.2 Statistical analysis

A total of 11 863 patients were recorded in eight Romanian maternity units in this clustered cross‐sectional study, aiming to assess the prevalence of episiotomy (prevalence ratio, PR). There was strong evidence of clustering within maternity units (intra‐class correlation coefficient −ICC = 0.07; 95% confidence interval: 0.03‐0.16 and *P* < 0.001). However, with the small number of clusters (maternity units), the estimation methods to correct for clustering may not perform well, illustrating that the random effects Poisson model for estimating the prevalence rate declined when there was a high ICC and with an increasing number of clusters. We used the random‐effects Poisson model to estimate the prevalence rate in univariate and multivariate models. For all fitted models, we used the adaptive Gaussian quadrature with eight integration points to approximate the log likelihood. STATA software version 13.1 was used for statistical analysis.

### 2.3 Ethical considerations

This study was approved by the institutional review board of the University of Medicine and Pharmacy of Targu Mures. Exemptions for a separate informed consent were obtained because the study was retrospective.

## 3 RESULTS

A total of 11 863 patients were recorded in eight maternity units of which 8475 (71.4%) had episiotomy (Table 1).

**Table 1:** Association between the prevalence of episiotomy and maternal and neonatal results

		Total Number of Patients	Prevalence of Episiotomy (%)^a^	Prevalence Ratio (95% CI)^b^	*P*‐Value^c^
Total		11 863	8475 (71.4)		
Parity	1	5470	5072 (92.7)	1	<.001
	2	3704	2713 (73.2)	0.80 (0.76‐0.83)	
	3	1265	441 (34.9)	0.38 (0.35‐0.42)	
	>3	1424	249 (17.5)	0.19 (0.17‐0.22)	
Gestational age (in weeks)	<30	84	45 (53.6)	1	<.001
	30‐33	265	147 (55.5)	1.03 (0.74‐1.44)	
	34‐37	1320	812 (61.5)	1.16 (0.86‐1.57)	
	>37	10 194	7471 (73.3)	1.42 (1.06‐1.90)	
Age group (in years)	<20	1218	982 (80.6)	1	<.001
	≥20	10 645	7493 (70.4)	0.84 (0.79‐0.90)	
Apgar score at 1 minute	0	17	17 (100.0)	1	.002
	1‐3	45	45 (100.0)	2.18 (1.25‐3.81)	
	4‐7	421	289 (68.6)	2.41 (1.48‐3.93)	
	>7	11 316	8124 (71.8)	2.46 (1.53‐3.96)	
Birth weight (in grams)	<1500	147	61 (41.5)	1	<.001
	1501‐2500	1192	683 (57.3)	1.34 (1.02‐1.74)	
	2501‐3500	8063	5941 (73.7)	1.75 (1.36‐2.25)	
	>3500	2461	1790 (72.7)	1.74 (1.35‐2.24)	
Use of oxytocin during the labour	No	6526	4274 (65.5)	1	.003
	Yes	5337	4201 (78.7)	1.08 (1.03‐1.14)	
Involvement of the ischiatic fosa	No	11 854	8467 (71.4)	1	0.48
	Yes	9	8 (88.9)	1.29 (0.64‐2.57)	
Anal sphincter involvement	No	11 823	8456 (71.5)	1	0.01
	Yes	40	19 (47.5)	0.56 (0.36‐0.89)	
Daytime of birth (in hours)	[8‐13]	1011	713 (70.5)	1	0.90
	[13‐21]	9084	6545 (72.0)	0.98 (0.89‐1.08)	
	[21‐8]	1768	1217 (68.8)	0.98 (0.90‐1.08)	

a *n* is the number of patients with episiotomy and % is the prevalence of episiotomy.

b 95% confidence interval.

c Wald test *P*‐value from the random effects Poisson model.

In the study group, 5.470 (46.1%) patients gave birth for the first time, 10.645 (89.7%) were over 20 years old, and 1.146 (9.7%) patients had a premature birth. Univariate analyses showed strong associations between episiotomy practice and parity, gestational age, age group, prematurity, birth weight and type of care provider (all *P* < .001 for all), Apgar score at 1 minute (*P* = .002), and use of oxytocin during labor (*P* = .003). There were non‐significant trends between episiotomy practice and maternity level (primary, secondary, tertiary) and also no evidence of association between episiotomy practice and time of birth.

We also investigated the occurrence of perineal lesions with respect to the practice of episiotomy practice in a univariate analysis for each of the following variables: labial tears, vaginal tears, anterior labial involvement, and cervical tear (Table 2).

**Table 2:** Association between need of suture and other perineal sutures with episiotomy practice

	Episiotomy	Not Episiotomy	Prevalence Ratio (95% CI)^a^	*P* Value^b^
Total	8.475	3.388		
Prevalence of need for suture *n* (%)^c^	8288 (97.8)	1112 (32.8)	2.99 (2.81‐3.18)	<.001
Prevalence of labial scar *n* (%)^c^	288 (3.4)	455 (13.4)	0.32 (0.27‐0.37)	<.001
Prevalence of vaginal scar *n* (%)^c^	46 (0.5)	255 (7.5)	0.09 (0.06‐0.12)	<.001
Prevalence of anterior involvement *n* (%)^c^	350 (4.1)	60 (1.8)	1.94 (1.48‐2.56)	<.001
Prevalence of cervical tear *n* (%)^c^	970 (11.4)	214 (6.3)	1.75 (1.50‐2.03)	<0.0001

a 95% confidence interval.

b Wald test *P*‐value from the random effects Poisson model.

c *n* is the number of patients with a given perineal lesion and % is the prevalence of the underlined

perineal lesion.

Episiotomy was strongly associated with labial tears, vaginal tears, anterior labial involvement, and cervical tears (all *P* < .001 for all). The necessity for sutures was three times greater in patients with episiotomy than in those without episiotomy (Table 3).

**Table 3:** Multivariable analysis showing the association between the episiotomy practice and type of surgeon, parity, Apgar score at 1 minute, and birth weight

	Covariates		Adjusted PR^a^	95% CI^b^	*P*‐Value^c^
Fixed effects component	Type of surgeon	Midwife	1		<.001
		Doctor	0.41	0.34‐0.51	
	Parity	1	1		<.001
		2	0.80	0.76‐0.84	
		3	0.39	0.36‐0.43	
		>3	0.20	0.17‐0.22	
	Apgar score at 1 minute	0	1		0.006
		1‐3	2.41	1.37‐4.23	
		4‐7	2.42	1.48‐3.97	
		>7	2.30	1.42‐3.74	
	Birth weight (in grams)	<1500	1		<.001
		2501‐3500	1.32	1.01‐1.73	
		1501‐2500	1.53	1.18‐1.98	
		>3500	1.62	1.24‐2.10	
Random effects component	Cluster level random intercept		Standard deviation estimate	SE^d^	
			0.43	0.12	

Likelihood ratio test testing whether the random intercept is needed: *P* < .001.

a Prevalence ratio.

b 95% confidence interval of the prevalence ratio.

c Wald test *P*‐value from random effects Poisson model.

d Standard errors of the estimate of the standard deviation of the random intercept.

The rates of anterior labial involvement and cervical tears were also moderately higher in patients with episiotomy than in those without. However, the rate of labial tears was 68% lower in patients with episiotomy, and the rate of vaginal tears was 91% lower in patients with episiotomy compared with those without episiotomy.

Among the available variables, parity, gestational age, age group, prematurity, birth weight, type of care provider, maternity level, use of oxytocin, and time of birth were used as potentially explanatory variables in a multivariate analysis. The choice of these explanatory variables was based on their antecedence to episiotomy potential utility to assess causal relationships that are not readily apparent in a cross‐sectional study. From the model selection procedure, gestational age, age group, prematurity, maternity level, use of oxytocin, and time of birth showed no evidence association with episiotomy practice after adjusting for all potential explanatory variables. We also investigated whether there was any interaction between prevalence of need of suture and parity and found no evidence (Table 4).

**Table 4:** Association between the prevalence of need for suture and parity

		Total Number of Patients	Prevalence of Need for Suture *n* (%)^a^	Prevalence Ratio (95% CI)^b^	*P*‐Value^c^
Total		118 63	9400 (79.2)		
Mother characteristics					
Parity	1	5470	5139 (94.0)	1	<.001
	2	3704	3094 (83.5)	0.89 (0.85‐0.93)	
	3	1265	714 (56.4)	0.60 (0.56‐0.65)	
	>3	1424	453 (31.8)	0.34 (0.31‐0.38)	

a *n* is the number of patients with episiotomy, and % is the prevalence of episiotomy.

b 95% confidence interval.

c Wald test *P*‐value from random effects Poisson model.

The prevalence of episiotomy was reduced by 60% when the care provider was a doctor instead of a midwife, after adjustment for parity, Apgar score at 1 minute, and birth weight. Compared with the first delivery, the prevalence of episiotomy was reduced by 20%, 61%, and 80% for the second delivery, third delivery, and subsequent deliveries, respectively. We tested for a linear trend in the association between episiotomy practice and parity and found strong evidence of nonlinearity. However, there was a monotonic decrease in the prevalence of episiotomy with increased parity. There were non‐significant trends between episiotomy practice and maternity level (Table 5).

**Table 5:** Association between the prevalence of episiotomy and maternity level

		Total Number of Patients	Prevalence of Episiotomy *n* (%)^a^	Prevalence Ratio (95% CI)^b^	*P* c
Total		11 863	8475 (71.4)		
Maternity level	Low	2789	1652 (59.2)	1	0.11
	Medium	4154	3131 (75.4)	1.22 (1.00‐1.50)	
	Academic	4920	3692 (75.0)	1.16 (0.97‐1.39)	

a *n* is the number of patients with episiotomy, and % is the prevalence of episiotomy.

b 95% confidence interval of the prevalence ratio. The prevalence ratio is adjusted for birth weight.

c Wald test *P*‐value from random effects Poisson model.

There was a strong association between episiotomy practice and Apgar score at 1 minute, after adjustment for parity, birth weight, and care provider (*P* < .001). Controlling for parity, Apgar score at 1 minute, and care provider, there was also a strong evidence of association between episiotomy practice and birth weight (*P* < .001). The prevalence of episiotomy was higher in patients whose newborn weighed 1500 to 2500 g, 2500 to 3500 g, or more than 3500 g compared with those whose newborn weighed less than 1500 g.

## 4 DISCUSSION

The Argentine Episiotomy Trial, the first randomized comparison of routine and selective episiotomy policies, was published in 1993.^10^ The study enrolled 2606 patients from eight maternity units in Argentina, and the authors concluded that “on the basis of current available evidence, a policy of routine episiotomy should be abandoned and rates above 30% cannot be justified.”^10^ More than 20 years after that landmark study Romania, the seventh largest country by population in the European Union, with around 200 000 babies born annually, has a 71.4% prevalence of episiotomy, according to the present study. Notably, the episiotomy rate at the first vaginal birth was 93% falling to 35% at the third vaginal birth. Thus, routine episiotomy is a common obstetrical practice in Romania, despite the good‐quality available evidence against its routine use ^2‐‐4,10^. This high rate of episiotomy is associated with an even higher rate of suturing, regardless of the parity; the likelihood of a woman leaving the hospital with an intact perineum after the first vaginal birth was only 5%. The apparent “protective” effect of the doctor compared with midwife must be interpreted with caution, because the midwife‐led birth is not common in Romania, where, according to regulations, a doctor must attend all deliveries.

Implementing a selective episiotomy policy in Romania could avoid a substantial number of surgical perineal repairs each year, an important gain for a country with frequent shortages in the medical system. In contrast to prior reports,^11^, ^12^, ^13^ we found no variation in episiotomy rate by time of birth, facility type (primary, secondary, tertiary), or geographical location.^14^, ^15^, ^16^, ^17^, ^18^ Webb and Culhane observed a clear temporal pattern in obstetric interventions,^11^ with episiotomies and other interventions to expedite delivery, such as operative vaginal delivery most likely to be performed during the day, and less likely at night. The authors suggested that “physicians may have multiple demands on their time during the day and may therefore feel more pressure to accomplish delivery more quickly than they do at night.”^11^ No such difference was found in our study, with similar rates of episiotomy throughout the day. The episiotomy rate was also similar across primary, secondary, and tertiary maternity units.

A recent American study reported a significant decline in the episiotomy rate from 2004 when episiotomy was performed in 25% of vaginal deliveries. In this analysis, the episiotomy rate decreased from 17.3% to 11.6% during the period 2006 to 2012. The study included 2 261 070 women who were hospitalized for a vaginal delivery at 510 hospitals, of whom 325 193 underwent episiotomy (14.4%).^17^ Demographic characteristics associated with the use of episiotomy included race (15.7% of white women vs 7.9% of black women) and insurance (17.2% of women with private insurance vs 11.2% with Medicaid insurance. Hospital factors (rural location and teaching status) were associated with lower episiotomy rates, but a wide variation among US hospitals was also noted, suggesting “non‐medical factors related to use of episiotomy” were also involved in the practice.^17^

The “protection” of the preterm fetus is also claimed by Romanian obstetric care providers to make the use of episiotomy very popular during preterm birth care. In our study, the episiotomy prevalence rate was lower among babies with a birthweight less than 1500 g. This finding may be associated with the low rate of complete antenatal corticotherapy in Romania among preterm neonates of the same group.^18^

Our study has some limitations. The cohort included both tertiary and community hospitals, and differences in practice across institutions were not accounted for. However, including data from a wide range of settings allowed us to investigate a more generalizable patient population. As our study was based upon maternity wards birth registries, it was also limited with respect to both the depth and accuracy of the clinical information available for analysis. Another limitation of this dataset and study design is the absence of information on morbidity and long‐term outcome in the population. Further studies, developed prospectively, can overcome these limitations.

## 5 CONCLUSION

In conclusion, routine episiotomy is the norm in Romanian maternity units with rates among the highest found in the medical literature. The likelihood of a primipara to leave a Romanian maternity unit with an intact perineum is less than 5%. Episiotomy use is primarily related to local professional practice, training during residency programs, and care provider preference, rather than to the real needs of individual women at the time of vaginal birth. Without knowing the “ideal” rate of episiotomy, efforts should be made to follow the best currently available evidence in order to reduce the over‐medicalization of normal vaginal birth, without putting our patients at risk for severe perineal laceration.

## CONFLICT OF INTEREST

None declared.

## AUTHOR CONTRIBUTIONS

A.P. and D.N. contributed equally to this article and should both be considered joint first authors. A.P. and D.N. wrote the draft manuscript. C.A.I., L.P., F.S., A.C., M.D., D.C., R.B., L.P., and D.N. contributing to further drafts and comments. All authors revised the draft and approved the final submitted article.

## References

[1] Jug Dosler A , Mivsek AP , Verdenik I , Skodic‐Zaksek T , Levec T , Petrocnik P . Incidence of episiotomy in Slovenia: the story behind the numbers. Nurs Health Sci. 2017;19(3):351‐357. 28631876 10.1111/nhs.12352

[2] Carroli G , Mignini L . Episiotomy for vaginal birth. Cochrane Database Syst Rev. 2009;3:CD000081. 10.1002/14651858.CD000081.pub2 PMC4175536 19160176

[3] Jiang H , Qian X , Carroli G , Garner P . Selective versus routine use of episiotomy for vaginal birth (review). Cochrane Database. 2017;2:CD000081. 10.1002/14651858.CD000081.pub3 PMC5449575 28176333

[4] Hartmann K , Viswanathan M , Palmieri R , Gartlehner G , Thorp J Jr , Lohr KN . Outcomes of routine episiotomy. A Systematic Review JAMA. 2005;293(17):2141‐2148. 15870418 10.1001/jama.293.17.2141

[5] Blondel B , Alexander S , Bjarnadottir RI , et al. Euro‐Peristat Scientific Committee. Variations in rates of severe perineal tears and episiotomies in 20 European countries: a study based on routine national data in Euro‐Peristat Project. Acta Obstet Gynecol Scand. 2016;95(7):746‐754. 26958827 10.1111/aogs.12894

[6] Ginod P , Khallouk B , Benzenine E , et al. Assessment of restrictive episiotomy use and impact on perineal tears in the Burgundy's perinatal network. J Gynecol Obstet Biol Reprod (Paris). 2016;45(9):1165‐1171. 27720515 10.1016/j.jgyn.2016.08.004

[7] Goldberg J , Purfield P , Roberts N , Lupinacci P , Fagan M , Hyslop T . The Philadelphia episiotomy intervention study. J Reprod Med. 2006;51(8):603‐609. 16967628

[8] Yamasato K , Kimata C , Huegel B , Durbin M , Ashton M , Burlingame JM . Restricted episiotomy use and maternal and neonatal injuries: a retrospective cohort study. Arch Gynecol Obstet. 2016;294(6):1189‐1194. 27439857 10.1007/s00404-016-4154-2

[9] Klein MC , Kaczorowski J , Robbins JM , Gauthier RJ , Jorgensen SH , Joshi AK . Physicians' beliefs and behaviour during a randomized controlled trial of episiotomy: consequences for women in their care. CMAJ. 1995;153(6):769‐779. 7664230 PMC1487268

[10] Argentine Episiotomy Trial Collaborative Group . Routine vs selective episiotomy: a randomized controlled trial. Lancet. 1993;342:1517‐1518. 7902901

[11] Webb DA , Culhane J . Time of day variation in rates of obstetric intervention to assist in vaginal delivery. J Epidemiol Community Health. 2002;56(8):577‐578. 12118047 10.1136/jech.56.8.577 PMC1732224

[12] Kaczorowski J , Levitt C , Hanvey L , Avard D , Chance G . A national survey of use of obstetric procedures and technologies in Canadian hospitals: routine or based on existing evidence? Birth. 1998;25(1):11‐18. 9534500 10.1046/j.1523-536x.1998.00011.x

[13] Webb DA , Culhane J . Hospital variation in episiotomy use and the risk of perineal trauma during childbirth. Birth. 2002;29(2):132‐136. 12000414 10.1046/j.1523-536x.2002.00173.x

[14] Howden NL , Weber AM , Meyn LA . Episiotomy use among residents and faculty compared with private practitioners. Obstet Gynecol. 2004;103(1):114‐118. 14704254 10.1097/01.AOG.0000103997.83468.70

[15] Goode KT , Weiss PM , Koller C , Kimmel S , Hess LW . Episiotomy rates in private vs resident service deliveries: a comparison. J Reprod Med. 2006;51(3):190‐192. 16674014

[16] Gossett DR , Dunsmoor SR . Episiotomy practice in a community hospital practice. J Reprod Med. 2008;53(10):803‐808. 19004409

[17] Friedman AM , Ananth CV , Prendergast E , D'Alton ME , Jason D , Wright JD . Variation in and factors associated with use of episiotomy. JAMA. 2015;313(2):197‐199. 25585333 10.1001/jama.2014.14774

[18] Suciu L , Pușcașiu L , Szabo B , Cucerea M , Ognean L , Oprea I . Mortality and morbidity of very preterm infants in Romania: how are we doing? Pediatr Int. 2014;56(2):200‐206. 24015920 10.1111/ped.12219

